# Dilatation of the Great Arteries in an Infant with Marfan Syndrome and Ventricular Septal Defect

**DOI:** 10.1155/2011/172109

**Published:** 2011-07-12

**Authors:** L. Rozendaal, N. A. Blom, Y. Hilhorst-Hofstee, A. D. J. Ten Harkel

**Affiliations:** ^1^Department of Pediatric Cardiology, Willem Alexander Children and Youth Center, Leiden University Medical Center, P.O. Box 9600, 2300 RC Leiden, The Netherlands; ^2^Department of Pediatric Cardiology, Emma Children's Hospital AMC, Academic Medical Center, 1105 AZ Amsterdam, The Netherlands; ^3^Department of Clinical Genetics, Leiden University Medical Center, 2300 RC Leiden, The Netherlands

## Abstract

We describe an infant presenting with contractures of the fingers, a large ventricular septal defect (VSD), and severe pulmonary artery dilatation. He had clinical and echocardiographic features of both neonatal or infantile Marfan syndrome (MFS) and congenital contractural arachnodactyly. After surgical VSD closure, the aortic root developed progressive dilatation while the size of pulmonary artery returned to normal limits. Eventually the diagnosis of MFS was confirmed by DNA analysis.

## 1. Introduction

In infancy the diagnosis of Marfan syndrome may be difficult, and clinical presentation and prognosis are variable. We present a special case of infantile Marfan syndrome with a congenital heart disease.

## 2. Clinical Presentation

**Figure 1:**
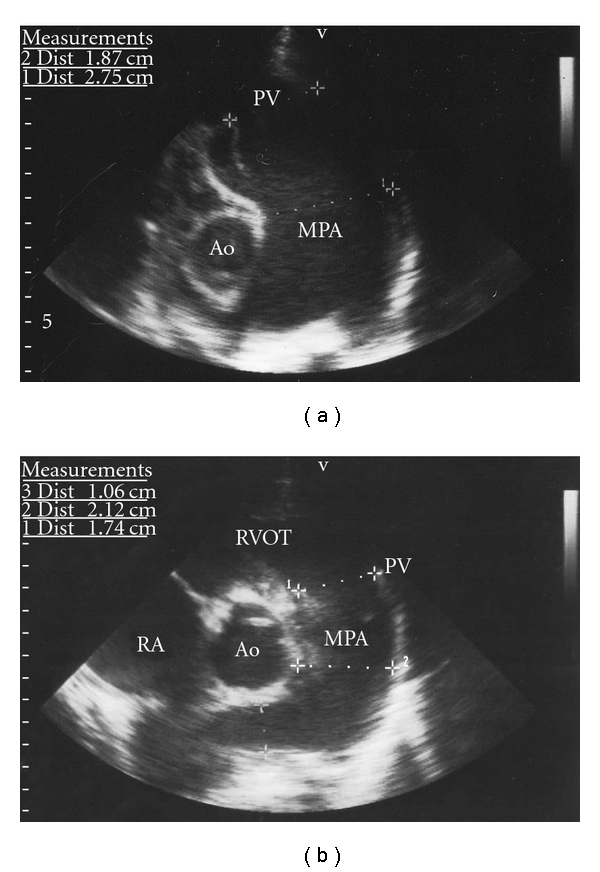
Parasternal short-axis view of the pulmonary valve (PV) and main pulmonary artery (MPA) of the patient preoperatively (a) and directly postoperatively (b). The severe dilatation of the PV (diameter 22 mm) and MPA (diameter 27 mm) diminished directly postoperatively, almost to normal limits (17.4 mm and 21.2 mm, resp.). RA: right atrium; RVOT: right ventricle outflow tract; Ao: aorta.

Our patient is a boy born to healthy parents (father is 37 years old, mother is 29 years old), who lives in Surinam. The pregnancy was uneventful, and he was born at a normal term. He had two healthy sisters of 6 and 9 years old. Family history was noncontributory. At the age of 4 months he developed heart failure and echocardiographic examination showed a large ventricular septal defect (VSD) and atrial septal defect (ASD) secundum type. The patient was sent to our institution for cardiac surgery at the age of 8 months. Physical examination showed a tall and dystrophic boy with a height of 75.0 cm (+1.5 SDS), weight of 5.5 kg (<−2.5 SDS), and an arm span of 72.0 cm (ratio arm span/height is 0.96). His target height is 187 cm (+0.4 SDS). He had no distinguished facial anomalies with a normal palate and normal ears. He had a pectus carinatum, arachnodactyly, and contractures of both proximal interphalanges of the third and fourth digits. He was dyspnoeic and tachypnoeic, and cardiac examination showed a hyperdynamic precordium, fixed splitting of the second heart sound, and no cardiac murmurs. Echocardiography showed a secundum-type ASD and a 17 mm muscular inlet VSD with a large left- to-right shunt causing pulmonary flow hypertension. The right atrium and right ventricle were dilated. Both mitral valve (MV) and tricuspid valve (TV) had a mild prolapse with a normal function. The pulmonary valve (PV), trunk, and branches were severely dilated with the diameter of the main pulmonary artery of 27.5 mm (Table 1 and Figure 1). The tricuspid aortic valve (AoV) showed normal function, and the aortic root diameter was dilated (Table 1). Surgical correction included primary closure of the ASD and closure of the VSD with a Gore-Tex patch. The postoperative course was uneventful with prompt recovery. Based on the clinical and echocardiographic features the differential diagnosis consisted of congenital contractural arachnodactyly (CCA) and neonatal or infantile Marfan syndrome (MFS). At that time no further gene testing was performed. After recovery the patient went home to Surinam, where the followup continued up to an age of 6 years.

**Figure 2:**
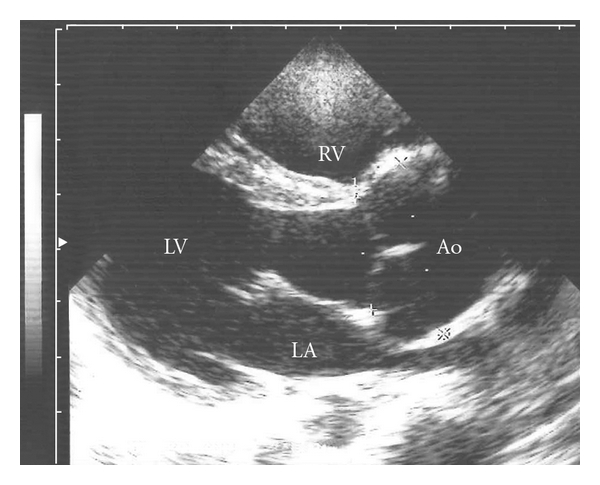
Parasternal long-axis view of left ventricle (LV) shows severe dilatation of the aortic root at the age of almost 4 years. Diameter of the aortic root is 32.2 mm. LA: left atrium; LV: left ventricle; RV: right ventricle; Ao: aorta.

During followup his height was excessive (SDS > 2.5), the contractures of the fingers were progressive, and he was wearing glasses because of myopia. Psychomotor development was normal. Interestingly, echocardiographic examinations showed significant decrease of diameters of the pulmonary valve and main pulmonary artery directly after operation which remained within normal limits during followup (Table 1, Figure 1). However, the diameter of the aortic root increased from 22 to 23.4 mm directly after operation and with progressive dilatation to 38.7 mm during followup (Table 1, Figure 2). Thus far, aortic valve function remained normal. The mild prolapse of the mitral valve remained stable with trivial mitral regurgitation. Because of progressive aortic root dilatation beta-blocking therapy was started at the age of 5 years.

At that time-gene testing was performed to further differentiate between infantile MFS and CCA. Using denaturing high-performance liquid chromatography (DHPLC), multiplex ligation-dependent probe amplification (MLPA kit P065/P066 v3), and DNA sequence-analysis in 2008, we found a frameshift mutation encoding a stop-mutation in the *FBN1* gene c.3396delA (p.Glu1133ArgfsX29) confirming the diagnosis of infantile Marfan syndrome.

## 3. Discussion

In infancy, the diagnosis of MFS can be differentiated in neonatal MFS, severe infantile MFS or early-onset form of MFS, and infants with a positive family mutation without major features at that moment. Differentiation is important because treatment and prognosis will be different, but this may be difficult [2–6]. Marfan syndrome is an autosomal dominant inherited disorder of connective tissue in which ocular, skeletal, cardiovascular, integumentary, pulmonary, and neurological features may be present in a highly variable degree [7–9]. The diagnosis is still a clinical one, based on fulfillment of diagnostic criteria [10]. It is caused by missense mutations in *FBN1* gene [11] on chromosome 15q21.1, the gene encoding fibrillin-1, a principle component of extracellular matrix microfibrils. A Marfan-like phenotype can be caused by mutations in the *TGFBR2* gene on chromosome 3, encoding TGF-beta receptor 2 [12]. In the classic form of Marfan syndrome, isolated occurrence is in 25–35% of the patients. Fibrillin indirectly controls TGF-beta activation, and dysregulation of TGF-beta may play a role in the pathophysiology of Marfan syndrome [13–23]. Main cardiovascular features are aortic root dilatation and/or aorta dissection and MV prolapse. Prognosis is mainly determined by progressive aortic root dilatation, potentially resulting in dissection and rupture. Prophylactic aortic root repair has raised the life expectancy by 30 years or more [24, 25].

Neonatal Marfan syndrome is a different group of patients with 50% mortality before the first year of life caused by heart failure. The definition of neonatal Marfan syndrome is an estimated diagnosis before 3 months of age, congenital pulmonary emphysema, severe atrioventricular valve (TV and MV) regurgitation in combination with congenital arachnodactyly, contractures, megalocornea, iridodonesis, rocker bottom feet, crumpled ears, and loose skin [3, 26, 27]. Especially congenital pulmonary emphysema and MV and/or TV insufficiency are very common (specific) in neonatal Marfan syndrome [3, 28]. The mutations are located in the central region of the *FBN1* gene (exons 24–32) and all are de novo mutations. However, mutations of patients with the classic form of Marfan syndrome have also been found in this region [4, 6, 26, 28–36]. 

Clinically our patient had severe infantile Marfan syndrome, early diagnosed because of the symptoms of a large ASD and VSD. We found a severely dilated pulmonary artery before operation, due to a large left-to-right shunt and pulmonary flow hypertension. Although pulmonary artery dilatation is a common finding in patients of all ages with MFS [37, 38], this case illustrates that the main pulmonary artery can dilate extremely due to increased pulmonary artery pressure and flow which appeared to be reversible after normalization of pulmonary artery pressure. In contrast, the aortic root showed progressive dilatation after shunt closure and improvement of left ventricular cardiac output. Congenital heart disease in MFS is rare and only a few cases have been described in literature [2, 4, 39–52]. Patent ductus arteriosus and atrial septal defect have been described in 4–6% of the patients [2, 4, 39, 41, 44, 47, 51] and may need surgical or interventional closure. The use of septal occluders should be considered with caution in the Marfan syndrome, because of risk of penetration [47]. An isolated VSD, without complex heart defect, as in our patient, has been reported in only 5 cases (infants and adults) in literature over the last 40 years [39–41, 46, 50, 52]. Coarctation of the aorta was reported once in a 10-year-old girl [45]. Three cases with complex congenital heart disease were described [42, 48, 49]. Congenital heart disease appears to be more common in Beals-Hecht syndrome or congenital contractural arachnodactyly (CCA), a dominantly inherited disorder of connective tissue characterized by multiple contractures, arachnodactyly, dolichostenomelia, scoliosis, and external ear anomalies, with mutations in *FBN2* gene [53, 54]. Skeletal features are almost the same as in Marfan syndrome, and dilatation of the aortic root and mitral valve prolapse, although less common, can also occur in CCA. The congenital heart defects described in CCA are very similar to those in MFS, including ASD, VSD, patent ductus arteriosus, coarctation, or interruption of the aorta [55, 56]. 

## 4. Conclusion

This special case of infantile MFS with congenital heart disease demonstrates severe dilatation of both great arteries with reversibility of pulmonary artery dilatation after normalization of the pulmonary artery pressure. It further illustrates that the clinical phenotypes of MFS and CCA show significant overlap and that genetic testing was required for final differentiation.

## Figures and Tables

**Table 1:** Aortic root diameters and pulmonary artery diameters during followup.

Age (yr)	Height cm (SDS)	Weight (SDS)	BSA (m^2^)	Aortic root mm (*z*-score)	PV mm (*z*-score)	MPA mm (*z*-score)
0.7	75.0 (+1.5)	5.5 (<−2.5)	0.34	22.0 (+8.6)	22.0 (+6.2)	27.5 (+8)
0.8	75.1 (+1.5)	5.6 (<−2.5)	0.34	23.4 (+9.2)	17.4 (+4.0)	21.2 (+6)
3.7	114.7 (>+2.5)	16.0 (<−2.5)	0.73	32.3 (+8.4)	20.0 (+1.9)	21.0 (+2)
5.7	129.0 (+2.5)	18.0 (<−2.5)	0.83	38.7 (+9.9)	19.0 (+0.9)	19.1 (+1)

BSA: body surface area; PV: pulmonary valve; MPA: main pulmonary artery. *z*-score according to Daubeney ([1], 284/id).

## Abbreviations

AoV: Aortic valve
ASD: Atrial septal defect
CCA: Congenital contractural arachnodactyly
*FBN1*: Fibrillin-1
*FBN2*: Fibrillin-2
MFS: Marfan syndrome
MPA: Main pulmonary artery
MV: Mitral valve
PV: Pulmonary valve
SDS: Standard deviation score
TV: Tricuspid valve
*TGFBR2*: TGF-beta receptor 2
TGF: Tissue growth factor
VSD: Ventricular septal defect.
